# Supplementary figures and images for: Experimental diets dictate the metabolic benefits of probiotics in obesity
Source: Gut Microbes. 2023 Mar 21;15(1):2192547. doi: 10.1080/19490976.2023.2192547 (PMC10038044; doi:10.1080/19490976.2023.2192547)

Figure S1

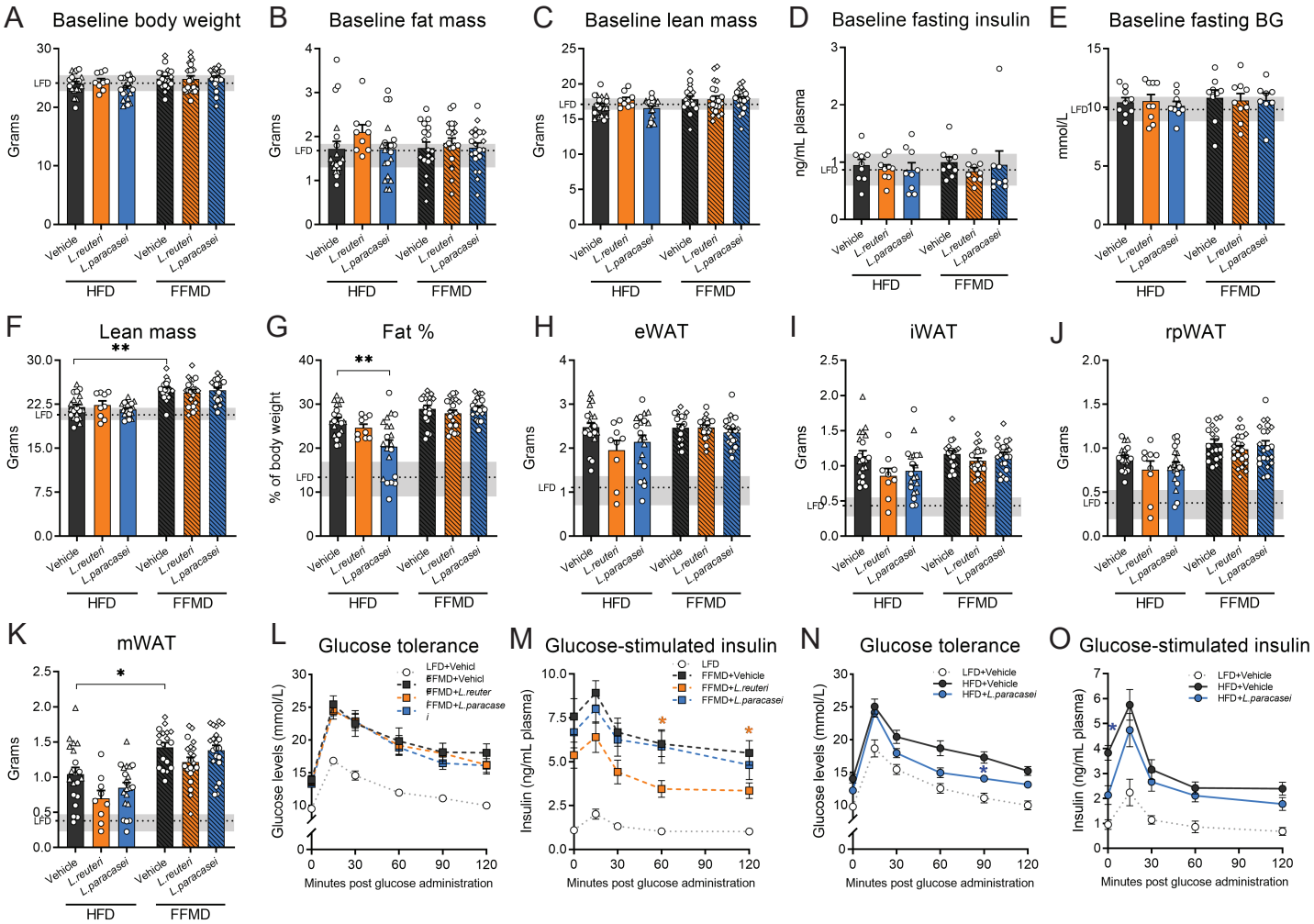

Figure S2

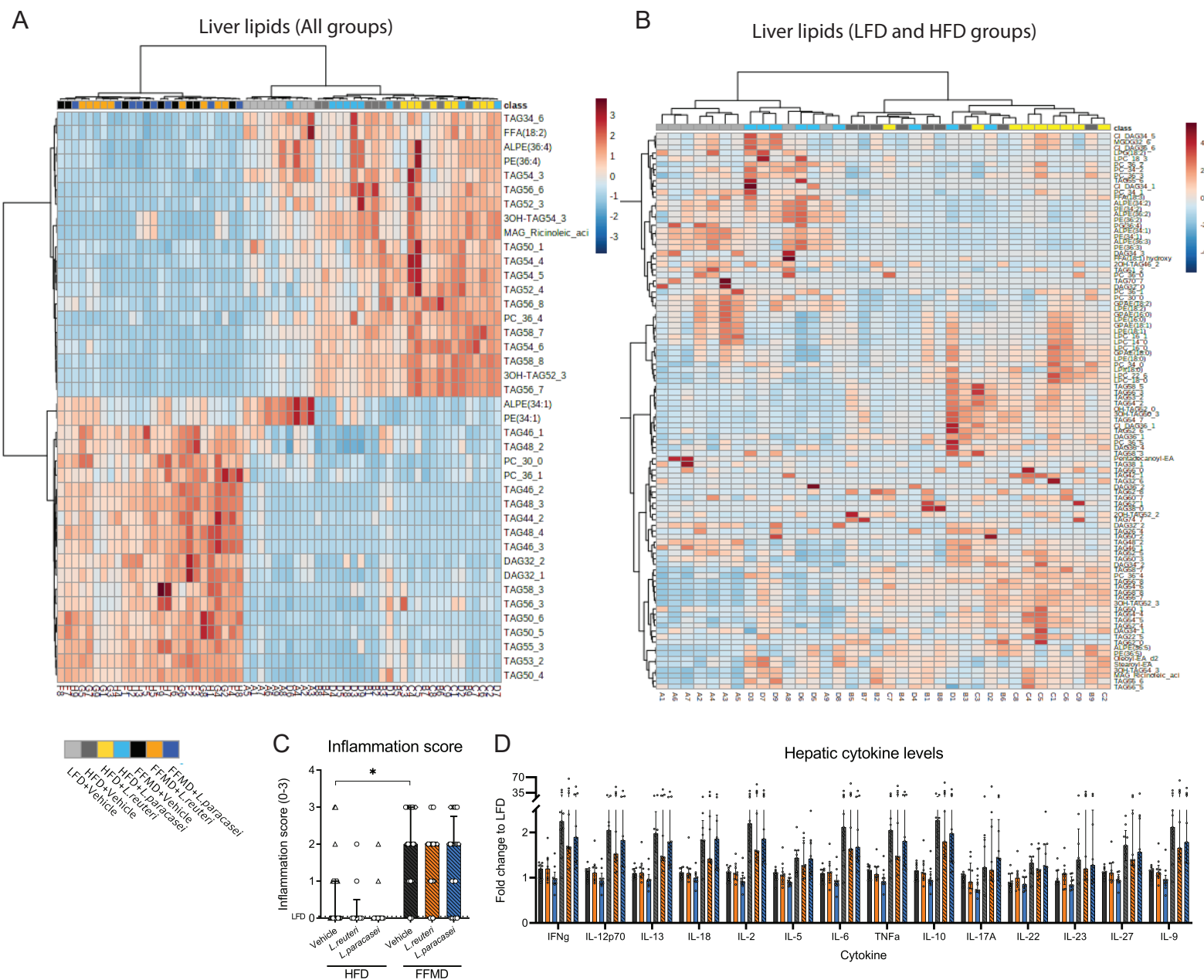

Figure S3

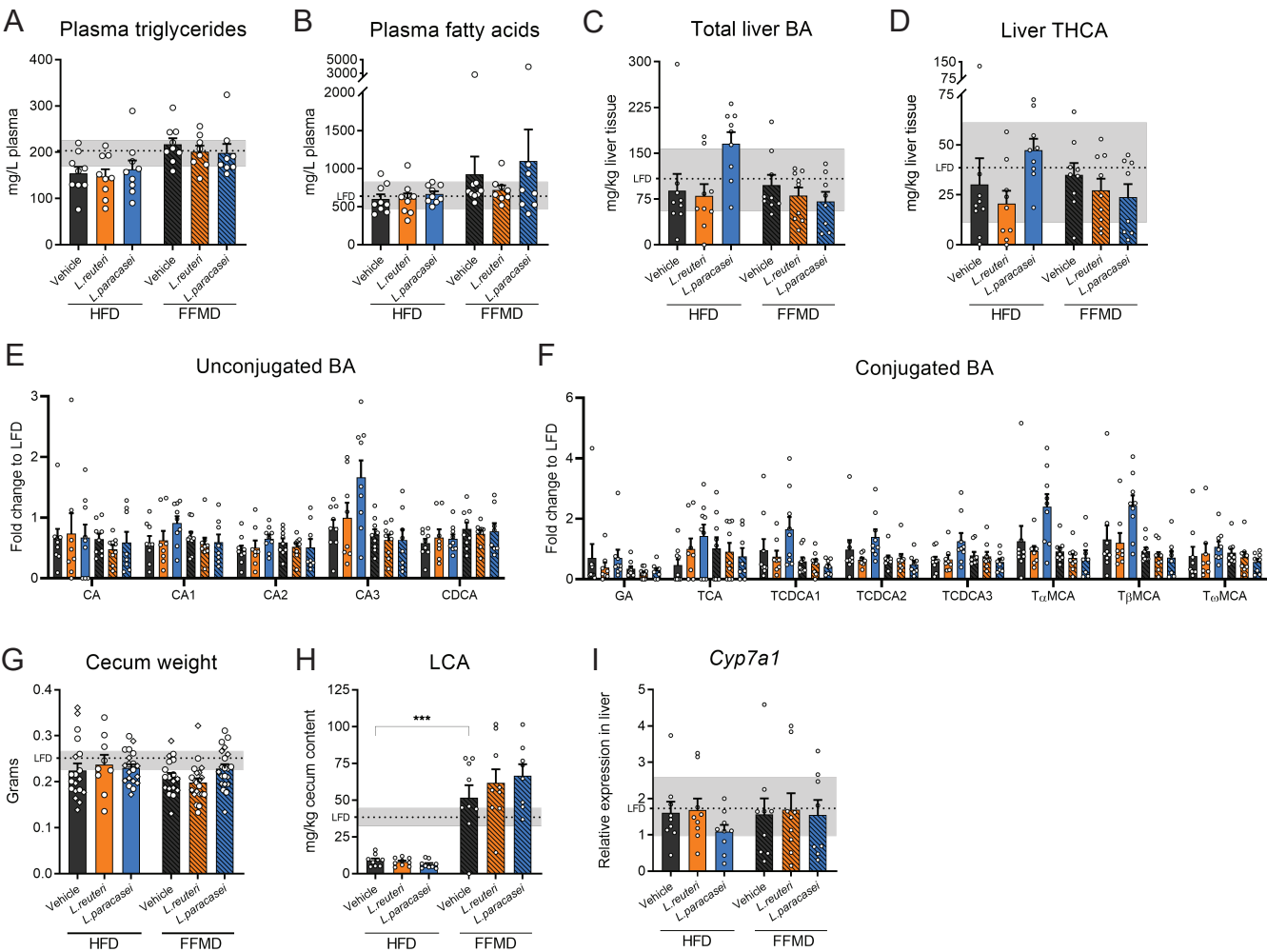

Figure S4

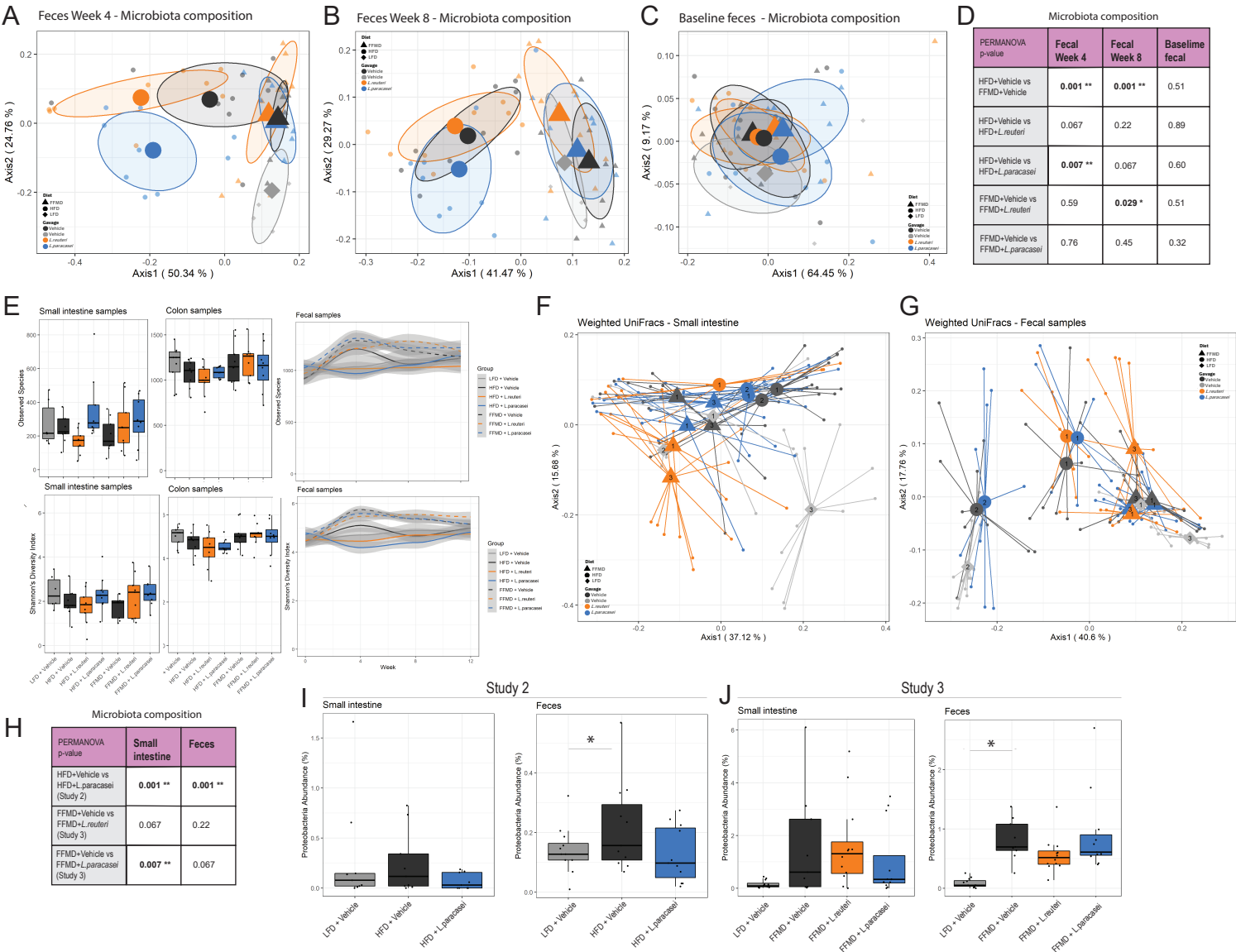

Supplement: Supplemental Material [file KGMI_A_2192547_SM2101.pdf]
